# Spatial transcriptomics technology in cancer research

**DOI:** 10.3389/fonc.2022.1019111

**Published:** 2022-10-13

**Authors:** Qichao Yu, Miaomiao Jiang, Liang Wu

**Affiliations:** ^1^ Beijing Genomics Institute (BGI)-Shenzhen, Shenzhen, China; ^2^ College of Life Sciences, University of Chinese Academy of Sciences, Beijing, China

**Keywords:** spatial transcriptomics (ST), tumor microenvironment, prognostic factor, spatial heterogeneity, tertiary lymphoid structure (TLS), tumor interface

## Abstract

In recent years, spatial transcriptomics (ST) technologies have developed rapidly and have been widely used in constructing spatial tissue atlases and characterizing spatiotemporal heterogeneity of cancers. Currently, ST has been used to profile spatial heterogeneity in multiple cancer types. Besides, ST is a benefit for identifying and comprehensively understanding special spatial areas such as tumor interface and tertiary lymphoid structures (TLSs), which exhibit unique tumor microenvironments (TMEs). Therefore, ST has also shown great potential to improve pathological diagnosis and identify novel prognostic factors in cancer. This review presents recent advances and prospects of applications on cancer research based on ST technologies as well as the challenges.

## Introduction

Cancer is the leading cause of death worldwide (1). A series of studies have shown that high cellular heterogeneity is one of the main causes that make cancer difficult to cure (2). Therefore, it is of vital importance to characterize the heterogeneity of tumor. Single-cell sequencing technologies, especially single-cell RNA sequencing (scRNA-seq), provides useful tools for uncovering cellular heterogeneity (3–6). However, they always lose spatial histology information while cell dissociation, hence difficult to characterize spatial cellular interactions and organization of tumors.

The spatial structure of tumors is closely related to tumorigenesis, progression, and treatment response. Histologically similar tumors from different regions are molecularly distinct and have different tumorigenicities, showing the association of tumor initiation and the spatial location (7). In addition, spatially adjacent cells have stronger interactions, which could functionally remodel the TME and promote tumor progression (8, 9) and evolution (10). The tumor cells at different positions may exhibit heterogeneous metastatic potential (5), while the site of lymphocyte infiltration usually indicates specific treatment response (11). Scientists have used immunohistochemistry (IHC) and immunofluorescence (IF) staining etc. technologies to locate the cells and proteins in flash-frozen or formalin fixed paraffin-embedded (FFPE) tissue sections. However, these approaches are low-throughput and can only label limited number of pre-selected proteins in a specific section, thus incapable of discovering the distribution of novel proteins or cell types. By contrast, ST technologies have much higher throughput and can capture the whole transcriptome, showing its power in constructing spatial cell atlas in embryo, brain, heart, etc. (12). In the meanwhile, an increasing number of studies used ST to profile spatial heterogeneity of cancers (13, 14). Currently, ST has been used to distinguish tumor and non-tumor tissues, special spatial areas such as tumor interface and tertiary lymphoid structures (TLSs) (15–17), and identify spatial-specific prognostic factors in cancer (18, 19).

In this article, we first made a brief introduction of ST techniques, and reviewed recent studies on cancer research using ST. Then, we proposed our insights into the challenges and prospects of applying ST into cancer research.

## Categories of ST

ST technology could be divided into two main categories according to detection methods, including imaging-based methods and sequencing-based methods (20). Imaging-based ST methods consist of in situ hybridization (ISH) and in situ sequencing (ISS) (**Table 1**). In ISH, RNA molecules from individual parts (or cells) within the tissue were achieved by hybridizing a labeled probe complementary to the target of interest. This technique was first used for visualizing gene expression in 1982 (21). Single-molecule RNA fluorescence in situ hybridization (smFISH) has a stronger and more robust signal compared with initial ISH (22). Afterwards, seqFISH (23), seqFISH+ (24), multiplexed error-robust FISH (MERFISH) (25), MERFISH+ (26) et al. further improved smFISH in the aspect of target throughput. Nevertheless, ISH-based methods are not transcriptome-wide, which always need prior knowledge to design probes and obstruct comprehensive expression analysis in a single experiment (27–29). In ISS, RNA molecules from a cell are sequenced directly in its tissue context. The first ISS technique was published in 2013, using padlock probes to target known genes (30). Later, BaristaSeq (31) and STARmap (32) improved sensitivity and/or number of detected genes. In short, most ISS-based ST techniques have subcellular resolution. However, they usually have a limited number of targeted genes or low detection efficiency (33), thus restricting their applications in the specific scenarios (**Table 1**).

The sequencing-based ST techniques include laser capture microdissection (LCM)-based methods and *in situ* barcoding (ISB)-based methods (**Table 1**). LCM-based methods, such as geographical position sequencing (Geo-seq) (34), TIVA (35) and NICHE-seq (36), utilize a laser beam to cut out specific tissue regions identified under a microscope (37, 38). Compared with initial LCM method, Geo-seq improved sensitivity but with lower resolution. TIVA can be performed on live cells but with low throughput, while NICHE-seq has higher throughput but not applicable to human samples. Generally, LCM-based ST techniques are labor-intensive and low-throughput, thus inapplicable of processing samples in batches. ISB-based ST techniques capture RNA molecules in situ, then perform cDNA sequencing ex situ. In terms of barcoding, it can be subdivided into two groups. The first group uses solid phase-based capture (SPBC) methods (13), and the tissue is transferred to a substrate bearing a pre-arranged set of DNA barcodes, which includes 10x Genomics Visium (39), Slide-seq (40), Slide-seq2 (41), HDST (42), Stereo-seq (43) etc. The second group, including NanoString digital spatial profiling (DSP) (44) and ZipSeq (45), uses selective barcoding methods, which means DNA barcodes are either collected from or delivered to selected tissue locations. ISB-based ST techniques have been used to study mouse olfactory bulb, gingival tissue, adult human heart tissue as well as multiple cancers (12). Most ISB-based techniques are transcriptome-wide with relative higher throughput, and some of them have subcellular resolution including Seq-Scope (46), HDST (42), APEX-seq (47), PIXEL-seq (48) and Stereo-seq (43) etc.

**Table 1:** Current ST technologies.

ST method	Category	Sample type	Resolution	Approach*	Advantages (+)/drawbacks (–)	Ref.
LCM-seq	LCM	Fresh-frozen	Cellular	WT, >16,000 genes in total	(+) Robust; full-length mRNA capture (-) Low throughput	(59)
TIVA	LCM	Live cells	Cellular	WT, >16,000 genes per cell	(+) Full-length mRNA capture from single live cells (-) Low throughput and limited analysis of clinical samples	(35)
tomo-seq	LCM	Fresh-frozen	Anatomical features	WT, ~23,000 gene in total	(+) Robust and high sensitivity; construction of 3D profiles (-) Limited applicability to clinical samples	(60)
Geo-seq	LCM	Fresh-frozen	Multicellular	WT, >8000 genes/20 cells	(+) Full-length mRNA capture; construction of 3D profiles (-) Low throughput	(34)
NICHE-seq	LCM	Live cells	Cellular/multicellular	WT, thousands of UMIs per cell	(+) High throughput (-) Limited to genetically engineered model organisms, so that not applicable for clinical samples currently.	(36)
PIC	LCM	Fresh-frozen/FFPE	Subcellular	WT, ~8000 genes/cell	(+) Relatively lower cost; Subcellular resolution (-) Limited field of view; Require manual choice of regions	(61)
immuno-LCM-RNAseq	LCM	Snap-frozen/RNAlater preserved	Multicellular	WT, >15,000 genes in total	(+) Compatible with low-quality samples; full-length RNA capture. (-) Low throughput	(62)
par-seqFISH	ISH	Cell cultures	Cellular	Targeted, 105 genes	(+) Applicable to bacteria. (–) Cannot be applied to human samples	(27)
smFISH	ISH	FFPE/Fresh-frozen	Subcellular	Targeted, several genes	(+) High sensitivity (–) Low throughput	(63)
seqFISH	ISH	Fresh-frozen	Subcellular	Targeted, 249 genes	(+) Subcellular resolution (-) Need costly equipment; Limited field of view	(29, 64)
MERFISH	ISH	Fresh-frozen	Subcellular	Targeted, 135 genes	(+) Highly multiplex; combined with IF for protein detection (-) Need costly equipment; Limited field of view	(25, 65)
smHCR	ISH	Fresh-frozen	Subcellular	Targeted, 40 probes	(+) mRNAs detection in thick (0.5mm) slices. (-) Low throughput; Limited field of view	(66)
RollFISH	ISH	FFPE	Subcellular	Targeted, several genes	(+) Applicable to FFPE samples (-) Low throughput	(67)
osmFISH	ISH	Snap-frozen	Subcellular	Targeted, 33 genes	(+) Large range of detectable gene expression levels (-) Relatively low throughput	(68)
RNAscope	ISH	FFPE	Subcellular	Targeted, 4 genes	(+) High sensitivity. Applicable to FFPE samples (-) Low throughput	(69)
seqFISH+	ISH	Fresh-frozen	Subcellular	Targeted, 10,000 genes	(+) Ultrahigh multiplex; Subcellular resolution (-) Limited field of view	(23)
SABER	ISH	Frozen	Subcellular	Targeted, 18,000 probes	(+) High sensitivity. Relatively low cost. (-) Limited field of view	(70)
Split-FISH	ISH	Fresh-frozen	Subcellular	Targeted, 317 genes	(+) High specificity. (-) Low throughput	(28)
DNA microscopy	ISH	Cell cultures	Cellular	Targeted, 10^6^ UMIs	(+) Relatively low cost. (-) Low throughput; Limited applicability to clinical samples	(71)
GeoMX WTA	ISH	FFPE	Cellular	Targeted, 18,190 genes	(+) Ultrahigh multiplex (-) Limited field of view; Require manual choice of regions	(72)
BOLORAMIS	ISH	Fresh-frozen	Subcellular	Targeted, 96 genes	(+) High sensitivity. (-) Low throughput	(73)
ISS using barcode padlock probes	ISS	FFPE/Fresh-frozen	Subcellular	Targeted, 31 transcripts	(+) Subcellular resolution; ability to detect SNVs (-) Limited number of target genes; low throughput	(30)
FISSEQ	ISS	FFPE/Fresh-frozen	Subcellular	WT, 8102 genes in total	(+) Subcellular resolution; Applicable to FFPE samples (-) Low sensitivity; limited field of view; low experimental throughput	(33)
BaristaSeq	ISS	Cell cultures	Subcellular	Targeted, several probes	(+) Relatively high amplification efficiency (-) Limited field of view; low experimental throughput	(31)
STARmap	ISS	FFPE/Fresh-frozen	Subcellular	Targeted, 1020 genes	(+) High sensitivity. Applicable to FFPE samples (-) Limited field of view; low experimental throughput	(32)
INSTA-Seq	ISS	FFPE/Fresh-frozen	Subcellular	Targeted, >820 genes	(+) High resolution; cDNA length up to 4750 nt (-) Limited field of view, low experimental throughput	(74)
BARseq	ISS	Fresh-frozen	Cellular	Targeted, 1.5 million barcodes	(+) High sensitivity and specificity (-) Limited field of view	(75)
HybISS	ISS	Fresh-frozen	Subcellular	Targeted, 119 genes	(+) Robust. High specificity. (-) Limited number of target genes	(76)
pciSeq	ISS	Fresh-frozen	Cellular	Targeted, 99 genes	(+) low misdetection rates. Relatively large field of view. (-) Limited number of target genes	(77)
sci-Space	ISS	Fresh-frozen	200 μm	WT, 1231 genes per cell	(+) larges field of view. (-) Low resolution.	(78)
ExSeq	ISS	FFPE/Fresh-frozen	Subcellular	WT/targeted, 3039/297 genes	(+) Support targeted and WT sequencing; multi-scale resolution; allow for AS detection. (-) Relatively low sensitivity	(79)
10x Genomics Visium	ISB	FFPE/Fresh-frozen	55 μm	WT, >20000 genes in total	(+) Robust; matched tools for downstream data analysis (-) multicellular resolution	(17, 39)
Slide-seq	ISB	Fresh-frozen	10 μm	WT, a median of 59 UMIs per cell	(+) Cellular resolution (-) Limited field of view; low sensitivity	(40)
HDST	ISB	Fresh-frozen	2 μm	WT, 63.5 UMIs per cell	(+) Ultrahigh resolution; high throughput (-) Limited field of view; low sensitivity	(42)
Slide-seqV2	ISB	Frozen	10 μm	WT, a median of 550 UMIs per cell	(+) Higher sensitivity than Slide-seq and HDST (-) Limited field of view	(41)
PIXEL-seq	ISB	Frozen	1 μm	WT, >1100 UMIs per cell	(+) Ultrahigh resolution; high sensitivity (-) Limited field of view; not accessible currently	(48)
Seq-Scope	ISB	Fresh-frozen	0.5-0.8 μm	WT, ~4700 UMIs per cell	(+) Ultrahigh resolution; ultrahigh sensitivity (-) Limited field of view	(46)
XYZeq	ISB	Fresh-frozen	Cellular	WT, a median of 1596 UMIs (629 genes) per cell	(+) Centimeter-scale field of view. (-) Relatively low sensitivity; Customized array	(80)
Stereo-seq	ISB	FFPE/Fresh-frozen	0.22 μm	WT, 1910 UMIs (792 genes) per cell	(+) Ultrahigh resolution; ultrahigh sensitivity; multi-scale field of views (from 0.5 to 174.24 cm^2^) (-) Customized array	(43)
ZipSeq	ISB	Live cells	Cellular	WT, 3550 genes per cell	(+) Single-cell resolution; high sensitivity (-) Costly reagents; Limited applicability to clinical samples	(45)
Nanostring DSP	ISB	FFPE/Fresh-frozen	Cellular	Targeted, 1412 genes or 44 proteins + 96 genes per cell	(+) FFPE compatible; multiomics spatial sequencing (-) Require manual choice of regions; Limited field of view;	(44)
APEX-seq	ISB	Live cells	Subcellular	WT, >25,000 transcripts in total	(+) Ultrahigh resolution; allow for AS detection. (-) Cannot be applied to human clinical samples; low throughput	(47)

*The number of genes/transcripts/probes/UMIs that can be detected. LCM, laser capture microdissection. ISH, in situ hybridization. ISS, in situ sequencing. ISB, in situ barcoding. WT, whole transcriptome. FFPE, formalin fixed paraffin-embedded. UMI, unique molecular identifier. IF, immunofluorescence. AS, alternative splicing.

Apart from the above-mentioned two ST categories, there also exists bioinformatic methods for reconstructing spatial positions of cells using scRNA-seq data. For example, novoSpaRc allows for *de novo* spatial reconstruction of single-cell gene expression with no inherent dependence on any prior information (49). Though many tools are available for the reconstruction of spatial positions of cells currently, their effectiveness remains to be validated in the future (50).

## ST provides new insights in cancer research

### Spatial heterogeneity of the tumor cell

**Figure 1:**
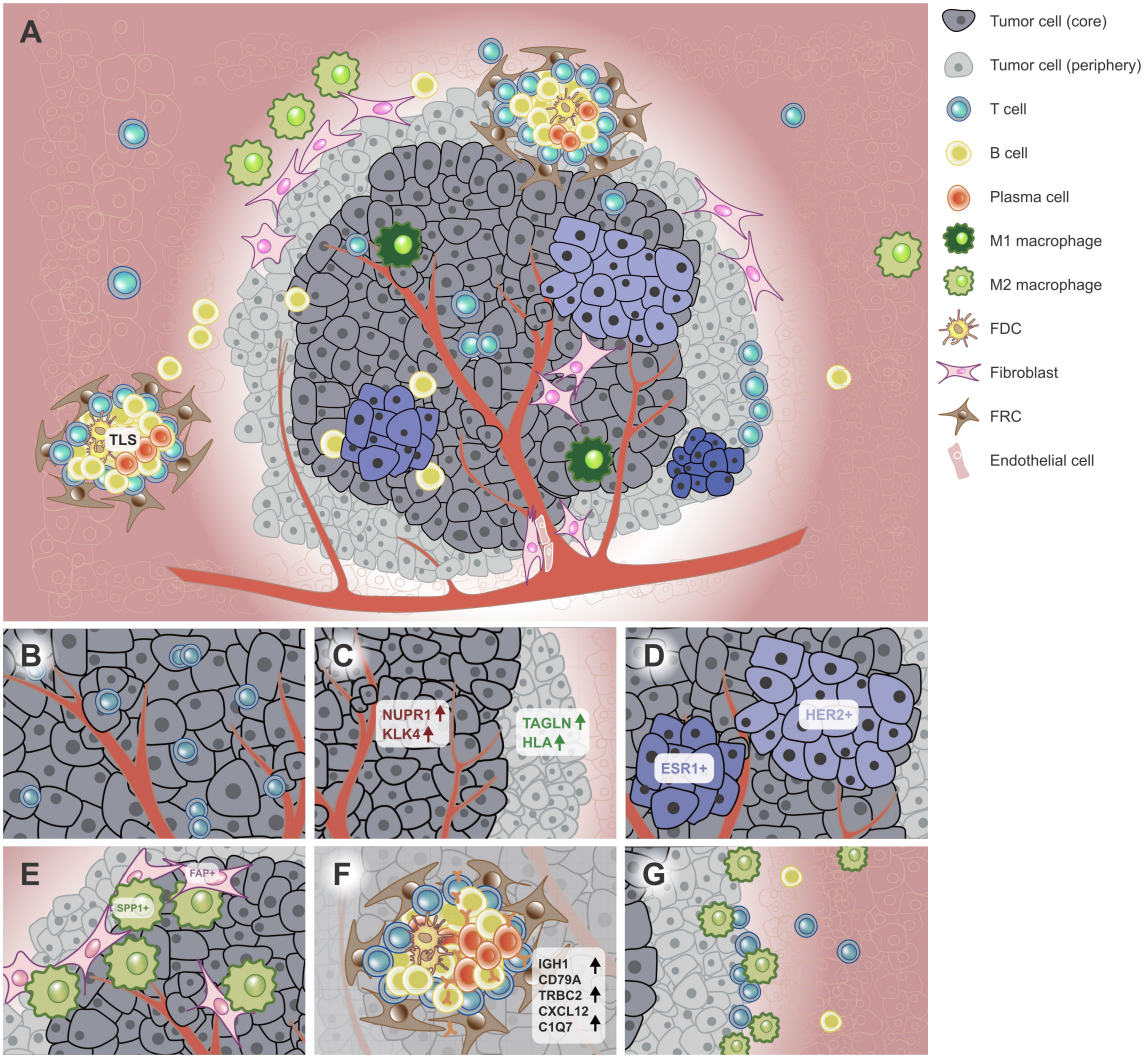
Spatial heterogeneity of the tumor. **(A)** ST techniques have been used to characterize the spatial architecture of tumors. For example, the tumor core (dark grey) and periphery (light grey) have different tumor cell subpopulations (light bule to dark blue). The TLS was found in or near the tumor, which aggregates diverse lymphoid cells. **(B)** CDH12+ tumor epithelial cells colocalized with exhausted CD8+ T cells in bladder cancer. **(C)** The tumor core and periphery had different signature genes in PC. **(D)** *HER2*+ and *ESR1*+ breast tumor subclones had mutually exclusive localizations in BC. **(E)** Tumor-specific *FAP*+ fibroblasts and *SPP1*+ macrophages colocalized in the CRC tissue. **(F)** TLSs have specific cell composition and signature genes. **(G)** The tumor interface has unique TME. Immune cells such as macrophages and NK/T cells were recruited to the interface and the region nearer to the interface enriched more immune cells in ICC. FDC, follicular dendritic cell; FRC, follicular reticular cell. PC, prostatic cancer. CRC, colorectal cancer. TME, tumor microenvironment. ICC, intrahepatic cholangiocarcinoma. BC, breast cancer. TLS, tertiary lymphoid structure.

Tumor tissues roughly consist of various cell types, including tumor cells, stromal cells, and immune cells (51) (**Figure 1A**). The differential cell composition induces the diversity and heterogeneity of TME. The heterogeneity confers different abilities of proliferation, immune resistance, immune escape, and survival etc. on tumor cells, which is reflected in many aspects, such as cell composition, gene expression pattern and cell spatial positions. Thus, tumor cells can be divided into several subpopulations in terms of genotype, phenotype, or spatial position.

Currently, neoplastic spatial heterogeneity has been reported in multiple malignant tumors, such as invasive micropapillary carcinoma (IMPC), gastric cancer (GC), glioblastoma (GBM), primary liver cancer and melanoma (19, 52–55) (**Table 2**). For instance, *ERBB2*, the receptor of HER2, differentially expressed in multiple tumor areas on the same ST slide (15). Further analysis showed these differentially expressed genes (DEGs) were associated with immune response, mitogenic programs and tumor invasion pathways, suggesting different areas may have differential abilities on tumor progression, invasion and immune resistance (39). Additionally, certain stress may induce certain new cell populations, which adapt to the specific TME. For instance, a functional subgroup was found in mouse pancreatic cancer model in a hypoxic microenvironment. Furthermore, the hypoxia-induced tumor tissues had less subpopulations and simplified functions (53). Intriguingly, though tumor cell subpopulations are spatially heterogeneous on the same slide, the transcriptional profile of each subpopulation is probably recurrent across tumor slides. Further analysis revealed these transcriptional programs were independent of cell-cycle states, thereinto reactive-hypoxia was associated with chromosomal alterations, indicating potential connection with genome instability (56). Moreover, tumor epithelial cells also exhibited specific colocalization with immune cell subpopulations (57, 58). In bladder cancer, CDH12+ epithelial cells colocalized with exhausted CD8+ T cells (57) (**Figure 1B**). In breast cancer (BC), tumor epithelial cells were negatively correlated with cancer-associated fibroblasts (CAFs), endothelial cells, B cells etc. (15). In sum, the diverse colocalization of tumor cell and other cell types inflects potential cell-cell interactions and the complex TME in tumors.

**Table 2:** ST-based cancer studies.

Spatial heterogeneity						
Cancer type	Species	Technology	Highlights	ST data access	Ref.
BC	human	Visium, scRNA-seq	HER2+ BC patients have common spatial expression signatures. Defined high-resolution cell state colocalization patterns	EGA: EGAD00001008031	(15)
BC	human	Spatial transcriptomics, scRNA-seq, ISH, smFISH	Developed 'spatial transcriptomics' technique and revealed spatial gene expression heterogeneity	SRA: PRJNA316587	(39)
BC	human	Visium, scRNA-seq, CITE-seq	Heterogeneous spatial distribution of tumor cell, immune cell, and stromal cell subpopulations.	https://doi.org/10.5281/zenodo.4739739	(11)
BC	human	Visium, snRNA-seq	Tumor cell subclusters with different features and origins are mapped in distinct tissue regions.	Not publicly available	(108)
TNBC	human	LCM	Proved combining LCM and RNA-seq on archived FFPE blocks is feasible and allows spatial transcriptional characterization of TME.	Array Express database: E-MTAB-8760	(109)
BC and oropharyngeal SCC metastasis	human	Visium, scRNA-seq	Identified colocalization patterns of immune, stromal, and cancer cells in tumor sections.	GEO: GSE158803	(83)
DCIS of the breast	human	Visium, target RNA-seq, scDNA-seq, WES	GATA3 dysfunction upregulates EMT and angiogenesis, followed by PgR downregulation.	DDBJ: JGAS00000000202	(110)
DCIS of the breast	human	LCM	Characterized spatial heterogeneity of DCIS using Smart-3SEQ.	SRA: PRJNA413176	(111)
IMPC	human	Visium	Characterized the spatial transcriptomic maps of IMPC and revealed extensive spatial heterogeneity associated with metabolic reprogramming.	GSA: HRA001442	(112)
Liver cancer/metastasis	human	smFISH, LCM, scRNA-seq	Characterized the spatial distribution and ligand–receptor interaction of cells in TME.	GEO: GSE146409	(113)
Liver cancer	human	Visium, WES, scRNA-seq	The ligand-receptor interactions at the tumor interface contribute to maintaining intratumor architecture.	GSA: HRA000437	(16)
ICC	human	stereo-seq, scRNA-seq, IF	Spatially characterized the immune microenvironment of tumor tissues, adjacent normal tissues, margin areas, and lymph nodes.	Not available	(19)
HCC	human	Visium	Revealed spatial intratumor heterogeneity and gene expression patterns of HCC.	Not available	(114)
PDAC	human	LCM, scRNA-seq, MS	Proposed subTME, which has regional relationships to tumor immunity, subtypes, differentiation, and treatment response.	EGA: EGAS00001002543	(84)
PDAC	human	Visium, scRNA-seq, IF	Characterized the spatial distribution of cells in TME and identified colocalization of inflammatory fibroblasts and cancer cells expressing a stress-response gene module.	GEO: GSE111672	(52)
PDAC	mouse	Visium, IHC, IF	The hypoxia group and the control group showed different positional characteristics and gene signatures.	Not available	(53)
GC	human	DSP, RNAScope, scRNA-seq, bulk RNA-seq	The expression level of *KLF2* in the tumor epithelial cell depends on its spatial location.	Not available	(58)
GC	human	RNAscope, scRNA-seq	Revealed the spatial distribution of the major cell types and *CCL2*-expressing endothelial cells and fibroblasts, indicating tumor invasion.	Not available	(91)
GC	human	DSP, targeted DNA-seq	Superficial subregion profiles were significantly different compared with matched deep subregions and LNM.	Not publicly available	(115)
Skin SCC	human	Visium, scRNA-seq, MIBI, WES	Characterized the TSK population, which localized to a fibrovascular niche and served as a hub for intercellular communication. Tregs colocalized with CD8 T cells in compartmentalized tumor stroma.	GEO: GSE144240	(92)
Melanoma	human	DSP, PickSeq, CyCIF	Recurrent cellular neighborhoods change significantly along a progression axis.	GEO: GSE171888	(55)
PC	human	Visium, WGS	Investigated tissue-wide spatial gene expression heterogeneity and identified gene expression gradients in stroma adjacent to tumor regions.	EGA: EGAS0000100300	(82)
Neuroblastoma	mouse, human	Visium, scRNA-seq, TCR repertoire	CD4+ and myeloid populations colocalized within the tumor parenchyma, while CD8+ T cells and B cells were peripherally dispersed.	SRA: PRJNA662418	(81)
GBM	human	Visium, MALDI, IMC, scRNA-seq, methylation array	Proposed five spatially distinct transcriptional programs. Immunosuppressive tumor-myeloid cell interactions are enhanced in segregated niches.	https://doi.org/10.5061/dryad.h70rxwdmj	(56)
Gliomas	human, mouse	ISH, scRNA-seq, WES	Characterized the spatial locations of TAMs and microglia.	http://glioblastoma.alleninstitute.org/	(54)
Bladder cancer	human	Visium, CODEX, snRNA-seq	CDH12-enriched cells express PD-L1 and PD-L2 and co-localize with exhausted T cells.	GEO: GSE171351	(57)
CRC	human	Visium, scRNA-seq, IF	Tumor-specific *FAP*+ fibroblasts and *SPP1*+ macrophages were colocalized. Their interaction may contribute to desmoplastic TME. Tumor-specific *FAP*+ fibroblasts are associated with colorectal cancer progression.	GSA: HRA000979	(90)
Cervical SCC	human	stereo-seq, snRNA-seq, IF	Characterized the spatial distribution of immune cells in cervical SCC. Certain tumors were surrounded by myofibroblasts, which was associated with growth and metastasis of tumors.	CNSA: CNP0002543	(93)
Colorectal cancer liver metastasis	human	Visium, scRNA-seq	Present a spatial atlas of colorectal liver metastasis and found the highly metabolically activated *MRC1*+ *CCL18*+ M2-like macrophages in metastatic sites.	http://www.cancerdiversity.asia/scCRLM/	(116)
Metastatic PC	human	DSP, bulk RNA-seq	Found a high level of intra-patient homogeneity with respect to tumor phenotype.	Supplementary of the original paper.	(95)
Melanoma LNM	human	Visium	Revealed a complex spatial intratumoral composition of melanoma metastases that was not evident through morphologic annotation.	Not available	(117)
**Special spatial area**						
BC	human	Visium, snRNA-seq	The *ERBB4*+ LumA cells scarcely existed in the tumor interface, whereas LumB cells were scattered throughout the tumor.	Not publicly available	(108)
BC	human	Visium, scRNA-seq	Proposed a method to identify putative TLSs.	EGA: EGAD00001008031	(15)
TNBC	human	LCM	CD8+ T cells of a patient are not located at the tumor core but rather at tumor margin.	Array Express database: E-MTAB-8760	(109)
Liver cancer	human	Visium, WES, scRNA-seq	Proposed a TLS-50 signature to locate TLSs.	GSA: HRA000437	(16)
ICC	human	stereo-seq, scRNA-seq, IF	Enrichment of immune cells, suppressive immune microenvironment and metabolic reprogramming of tumor cells were identified in the invasive fronts of tumor.	Not available	(19)
Liver cancer/metastasis	human	smFISH, LCM, scRNA-seq	Higher abundance of immune cell types, specifically T cells and SAMs, in the tumor border.	GEO: GSE146409	(113)
Melanoma	human	DSP, PickSeq, CyCIF	A spatially restricted suppressive environment forms along the tumor-stromal boundary when tumors are locally invasive.	GEO: GSE171888	(55)
Melanoma	zebrafish, human	Visium, scRNA-seq, snRNA-seq, IF	Identified a distinct interface cell state where the tumor contacts neighboring tissues.	GEO: GSE159709	(107)
Skin SCC	human	Visium, scRNA-seq, MIBI, WES	Tumor leading edges were enriched with tumor-specific TSK cells and basal tumor cells.	GEO: GSE144240	(92)
PDAC	mouse	Visium, IHC, IF	A cell subgroup located at the invasive front showed a higher proliferative ability under hypoxia.	Not available	(53)
GBM	human	Visium, scRMA-seq. Bulk RNA-seq, IF	A *HMOX1*+ myeloid cell subpopulation, spatially located at the TME interface, contributes to immunosuppressive TME.	https://osf.io/4q32e/	(97)
RCC	human	Visium, IHC,IF, bulk RNA-seq	In situ B cell maturation toward plasma cells in TLSs. Tumor cells are labeled by locally produced IgG.	GEO: GSE175540	(17)
**Cancer treatment response**						
PDAC	mouse	Visium, IF	Identified a potential treatment target for PDAC.	Not available	(53)
PDAC	human	LCM, scRNA-seq, MS	SubTMEs execute distinct tumor-promoting and chemoprotective functions.	EGA: EGAS00001002543	(84)
PDAC	human	DSP, snRNA-seq	In the increasingly-adopted NAT context, classical-like phenotypes in malignant cells were depleted.	Controlled access	(118)
GBM	human	RNAscope, targeted DNA-seq	Inhibitory molecules and infiltration increased after CART-EGFRvIII infusion, compared to pre-CART-EGFRvIII infusion tumor specimens.	Not publicly available	(119)
RCC	human	Visium, IHC, IF, bulk RNA-seq	Patients with IgG-labeled tumor cells have high response rate to ICI and prolonged PFS.	GEO: GSE175540	(17)
BC	human, mouse	Visium, scRNA-seq	Neutralization of TGF-β leads to remodeling of CAF dynamics, greatly reducing the frequency and activity of the myofibroblast subset.	Not publicly available	(120)
Head and neck SCC	human	RNAscope	PD-L1 and PD-L2 positivity significantly predicted clinical response to pembrolizumab on combined tumor, stromal and immune cells.	Not available	(121)
Lung cancer	mouse	Visium, Perturb-map, IMC, CyTOF	Tgfbr2 KO on cancer cells promotes TME remodeling and immune exclusion; Socs1 KO made the tumors more responsive to PD-L1 blockade.	GEO: GSE193460	(89)
CRC	human	Visium, scRNA-seq, IF	High infiltration of *FAP*+ fibroblasts and *SPP1*+ macrophages correlated with immunotherapy resistance.	GSA: HRA000979	(90)
Colorectal cancer liver metastasis	human	Visium, scRNA-seq	Observed fundamental remodeling of cellular compartment after NAC treatment. PD/SD tumors and PR tumors had different immune cell changes after NAC.	http://www.cancerdiversity.asia/scCRLM/	(116)
Bladder cancer	human	Visium, CODEX, snRNA-seq	CDH12-enriched tumors define patients with poor outcome following surgery with or without NAC, whereas they exhibit superior response to ICI treatment.	GEO: GSE171351	(57)
Ovarian carcinoma	human	Visium	Excellent and poor responders show different spatial composition of TME.	GEO: GSE189843	(122)
PC	human	Visium, scRNA-seq, scATAC-seq, FAIRE-seq	Treatment-persistent cells with high metastatic potential interspersed within the primary tumors before treatment.	EGA: EGAS00001000526	(123)
**Clinical application**						
**Diagnosis**						
BC	human	Visium, scRNA-seq, CITE-seq	Developed scSubtype for BC subtype classification using scRNA-seq data.	https://doi.org/10.5281/zenodo.4739739	(11)
BC	human	ISS	OncoMap could spatially reveal intratumoral heterogeneity with regard to tumor subtype, which supports the identification of novel therapeutical targets and refine tumor diagnostics.	Not available	(124)
BC	human	Visium	ST-based annotation showed high coincidence with the expert pathologist annotation for DCIS and IDC.	No raw ST data	(125)
DCIS of the breast	human	Visium, targeted RNA-seq, scDNA-seq, WES	Propose a critical marker for a new DCIS classification approach.	DDBJ: JGAS00000000202	(110)
IMPC	human	Visium	The pathologists and ST data were consistent in their annotation of the tumor tissues.	GSA: HRA001442	(112)
PC	human	Visium, scRNA-seq, scATAC-seq, FAIRE-seq	Identified benign epithelium and adenocarcinoma using ST data.	EGA: EGAS00001000526	(123)
PC	human	Visium, WGS	Compared to pathologist annotations, ST-based annotation delineates the extent of cancer foci more accurately.	EGA: EGAS0000100300	(82)
**Prognosis factors**						
Liver cancer	human	Visium, WES, scRNA-seq	Higher TLS-50 score was significantly associated with a better prognosis.	GSA: HRA000437	(16)
ICC	human	stereo-seq, scRNA-seq, IF	The damaged states of hepatocytes with overexpression of *SAA* in invasive fronts were associated with worse prognosis.	Not available	(19)
HCC	human	Visium	High expression of *CCL15* and *CD163* respectively predicts poor prognosis of HCC patients. *CCL19* and *CCL21*, sharing similar spatial expression patterns, indicate a good prognosis.	Not available	(114)
Bladder cancer	human	ST, CODEX, snRNA-seq	Patients stratification by tumor CDH12 enrichment offers better prediction of outcome than currently established bladder cancer subtypes.	GEO: GSE171351	(57)
GC	human	DSP, RNAScope, scRNA-seq, bulk RNA-seq	*INHBA* and *FAP* were coexpressed. CAFs with high expression of *INHBA-FAP* were associated with poor prognosis.	Not available	(58)
GC	human	RNAscope, scRNA-seq	Deep-layer endothelial cells and fibroblasts contributed to poor clinical outcomes.	Not available	(91)
GC	human, mouse	LCM	A stromal gene signature was associated with poor disease outcome, and HSF1 regulated the signature.	GEO: GSE162301, GSE165211	(126)
IMPC	human	Visium	The high expression levels of the *SREBF1* and *FASN* indicated a poor prognosis.	GSA: HRA001442	(112)
PC	human	Visium, scRNA-seq, scATAC-seq, FAIRE-seq	High PROSGenesis score was associated with good prognosis.	EGA: EGAS00001000526	(123)
Skin SCC	human	Visium, scRNA-seq, MIBI, WES	High expression of TSK-specific genes *ITGB1* and *PLAU* correlated with significantly reduced PFS.	GEO: GSE144240	(92)
Cervical SCC	human	stereo-seq, snRNA-seq, IF	Myofibroblasts were associated with poor survival.	CNSA: CNP0002543	(93)
CRC	human	Visium, scRNA-seq, IF	High infiltration of *FAP*+ fibroblasts and *SPP1*+ macrophages correlated with worse prognosis.	GSA: HRA000979	(90)
PDAC	mouse	Visium, IF	Identified genes associated with good or poor prognosis.	Not available	(53)

SRA, Short Read Archive. GSA, Genome Sequence Archive. CNSA, China National GeneBank Sequence Archive. EGA, European Genome-Phenome Archive. DDBJ, the DNA Data Bank of Japan. IDC, invasive ductal carcinoma. DCIS, ductal carcinoma in situ. IMC, imaging mass cytometry. MS, Mass spectrometry. SCC, squamous cell carcinoma. IMPC, invasive micropapillary carcinoma. BC, breast cancer. TNBC, triple-negative breast cancer. GC, gastric cancer. GBM, glioblastoma. CRC, colorectal cancer. PDAC, pancreatic ductal adenocarcinomas. PC, prostatic cancer. LNM, lymph node metastasis. HCC, hepatocellular carcinoma. ICC, intrahepatic cholangiocarcinoma. RCC, renal cell carcinoma. ISS, in situ sequencing. ISH, in situ hybridization. LCM, laser capture microdissection. IF, immunofluorescence. smFISH, single-molecule RNA fluorescence in situ hybridization. MIBI, multiplexed ion beam imaging. CODEX, co-detection by indexing. CyTOF, cytometry by time of flight. WES, whole-exome sequencing. WGS, whole-genome sequencing. DSP, digital spatial profiling. IHC, immunohistochemistry. TCR, T cell receptor. FFPE, formalin fixed paraffin-embedded. TSK, tumor-specific keratinocyte. TAMs, tumor-associated macrophages. TLSs, tertiary lymphoid structures. SAMs, scar-associated macrophages. CAFs, cancer-associated fibroblasts. NAT, neoadjuvant treatment. ICI, immune checkpoint inhibitor. NAC, neoadjuvant chemotherapy. PFS, progression free survival. KO, knockout. PD, progressive disease. SD, stable disease, PR, partial response.

Tumor cells also showed different gene expression profiles in tumor core and periphery. Compared with tumor core, the tumor peripheral area is nearer to adjacent normal tissues, thus it has different TME **(** **Figure 1A**). In a neuroblastoma mouse model, a tumor cell cluster, which was most enriched at the tumor core, expressed more cancer-associated genes than that dispersed all over the tumor area (81). In prostatic cancer (PC), *TAGLN* (tumor suppressor) and *HLA* had higher expression in the periphery, whereas *NUPR1* and *KLK4* etc. were expressed higher in the tumor core (82) (**Figure 1C**). These findings jointly indicate the tumor core probably is more malignant than the periphery.

A series of ST-based studies showed tumor subpopulations are spatially mutually exclusive on the same slide (**Figure 1A**). In BC, the tumor area with high signatures of epithelial-mesenchymal transition (EMT), interferon (IFN) and major histocompatibility complex (MHC) was negatively correlated with that with high signature of proliferation (11). Another study revealed *HER2*+ and *ESR1*+ breast tumor clones had mutually exclusive localizations (83) (**Figure 1D**). In primary pancreatic ductal adenocarcinomas (PDAC), *TM4SF1*+ tumor cells had mutually exclusive spatial locations with *S100A4*+ tumor cells (52). Another study identified three recurrent sub-TME phenotypes within the same tumor tissue in PDAC, which also showed clear boundaries with each other (84). The mutually exclusive locations of tumor cell subpopulations suggest different clonal origins and potential competition across tumor areas (39).

### Spatial heterogeneity of microenvironment

The stromal cell is an important component of TME, which is associated with tumor growth, progression, immunosuppression and metastasis (85–89). ST has uncovered a series of spatial distribution preference of stromal cells in TME. For example, in a lung cancer mouse model, loss of Tgfbr2 resulted in a remodeling of the stroma and induced tumor development (89).

Among stromal cells, fibroblasts showed the most prominent spatial colocalization features in multiple cancer types. In bladder cancer, fibroblasts were observed resided in close proximity to CDH12+ epithelial cells (57). In colorectal cancer (CRC), tumor-specific *FAP*+ fibroblasts and *SPP1*+ macrophages colocalized in the tumor area, which were proved contributed to desmoplastic TME (90) (**Figure 1E**). In PDAC, fibroblasts and terminal ductal cell populations were significant enrichment in tumor areas, suggesting ductal cells in the cancer region may express hypoxia-response genes due to low oxygen content. In diffuse-type GC, *CCL2*+ fibroblasts and endothelial cells were enriched in the deep invasive layer of GC compared with the superficial layer, suggesting a greater ability of tumor invasion (91). In addition, CAFs also exhibited well-preserved colocalization patterns with endothelial cells and perivascular cells (15), which is consistent with a previous study in cutaneous squamous cell carcinoma SCC (92). Interestingly, in cervical SCC, CAFs were enriched around certain tumor areas. Compared with tumors without surrounded by CAFs, CAF-surrounded tumors were more active in metabolism and cell growth and downregulated cellular adhesion, apoptosis, and immune response, suggesting a supportive TME for tumor progression and metastasis (93). On the other hand, fibroblast subpopulations may have mutually exclusive locations. For example, Wu et al. found spatially negative correlation between myofibroblast-like CAFs and inflammatory-like CAFs in estrogen receptor positive and triple-negative breast cancer (TNBC) samples (11). This finding was also observed in a HER2+ breast tumor dataset, suggesting a conserved relationship across BC subtypes (15).

The immune cell infiltration is frequently observed in tumor tissues, which is closely associated with treatment response (94). However, not all immune cells can infiltrate into tumors (81), especially in metastases (95). ST-based studies have shown the immune cell has a specific preference on the spatial distribution as well. For instance, in a lung cancer mouse model, immune cells were more concentrated in the outer area of tumors, particularly CD4+ and CD11c+ cells. Intriguingly, when Irf1 or Socs1 was knocked out, immune cells were scattered throughout the tumor (89). As we know, macrophages can be roughly divided into M1 macrophage and M2 macrophage in terms of encouraging or decreasing inflammation (96). M1-like macrophage and M2-like macrophage appeared to have mutually exclusive locations in PDAC. Of note, a M2-like macrophage subpopulation was observed colocalized with proliferating cancer cells (83, 90). The M2-like macrophages were most enriched in the ducts, whereas the M1 macrophage were enriched in the stroma and cancer regions (52). Similarly, in BC, two macrophage subpopulations, which were outside of the conventional M1/M2 classification, displayed a modest negative spatial correlation (11). In addition, macrophage subpopulations also colocalized with T cell subpopulations in BC and neuroblastoma (15, 81). Further analysis revealed the colocalization might be associated with immune activation and tumorigenesis. In GBM, the colocalization of immune cells and stromal cell subpopulation was associated with immunosuppressive microenvironment (54, 97). Additionally, the presence of certain tissues may also influence the distribution of T cell subtypes. In liver cancer, intact continuous fibrous capsule indicated significant decrease of exhausted T cells and downregulated immune checkpoint genes, suggesting it may act as a barrier preventing the infiltration of immune cells (16). In addition, *SPP1*+ tumor-associated macrophages (TAMs) were colocalized with proliferating cancer cells (83), which is consistent with a recent study in CRC (90).

### Special spatial structures in TME

#### Tertiary lymphoid structures

TLSs, sometimes also known as tertiary lymphoid organs or ectopic lymphoid structures, are organized aggregates of lymphoid cells that arise postnatally in nonlymphoid tissues, such as tissues subjected to chronic inflammation and cancers (98). They are characterized by an inner zone of CD20+ follicular B cells surrounded by CD3+ T cells. Recently, more evidence supported the important function of TLS in delaying or promoting cancer progression (98–100).

TLS was mostly found in para-tumor tissues (16). However, it also exists in tumor, leading-edge and para-tumor areas (19, 98) (**Figure 1A**). Previous studies generally identify TLS by multiple IHC or multiplex IF staining (101–103), which is accurate but labor-consuming and low-throughput. Mature TLSs are characterized by the presence of a germinal center containing T follicular helper (Tfh) cells and follicular dendritic cells in close contact with B cells (104). TLS could be identified by certain gene signatures or cell population aggregates using ST (**Figure 1F**; **Table 2**). For example, in BC, Andersson et al. evaluated the degree of B- and T-cell colocalization by spot deconvolution, ergo potentially constituting parts of a TLS which they called TL-like structure. At last, they proposed a TLS signature with 171 genes for TLS prediction (15). In renal cell carcinoma (RCC), Meylan et al. proposed a TLS signature with 29 genes (17). A similar gene signature was also proposed in liver cancer (16). Of note, in BC, researchers found a negative correlation between B cells and plasma cells, and colocalization of B cells and T cells (15), which probably suggested the presence of TLS.

#### Tumor interface has unique ecosystem

The tumor interface, the invasion front for tumor cells to expand, is a critical region to uncover the tumor invasion and progression (5, 105–107). The intermediate zone between the tumor tissue and the non-tumor tissue has unique microenvironment, thus existing unique characteristics in cell-cell interaction, cell composition and immune states (**Table 2**).

The tumor interface exhibits a transitional state between the tumor and adjacent non-tumor tissues. In a zebrafish model of melanoma, a distinct cell cluster at the interface was found histologically resembled the microenvironment, but transcriptionally resembled tumor, and may function as a bridge for interaction between tumor and neighboring tissues (107). This unique interface was also observed in human melanoma (107). In primary liver cancer, *PROM1*+ and *CD47*+ cancer stem cell (CSC), which was scattered in tumor areas, was found to be gradually increased from leading-edge to tumor to portal vein tumor thrombus, and closely related to TME remodeling and tumor metastasis. The unique metabolic features at the interface, where may occur sudden decrease or increase of certain hallmark pathways, suggest it may serve as a buffer between tumor and normal regions (16).

The tumor interface has specific cell compositions. In cutaneous SCC, a tumor-specific keratinocyte (TSK) subpopulation located at the leading edges functioned as a hub for intercellular communication, which is probably due to short distance between the interface and tumor or normal tissues (92). In BC, LumA tumor cells scarcely existed in the interface of tumor, whereas LumB cells were scattered throughout the tumor tissue, suggesting heterogeneous spatial distribution of tumor cell subpopulations (108). In a mouse pancreatic cancer model, a tumor cell cluster upregulated *LDHA* (a hypoxia-related gene) in the interface area compared with that in the tumor center and had stronger ability for survival and invasion (53).

The tumor interface has unique immunosuppressive TME as well. In intrahepatic cholangiocarcinoma (ICC), macrophage and NK/T cells were enriched close to the boundaries. Of note, immune cells were recruited to the borderline from the tumor side, and the region nearer to the borderline enriched more immune cells, showing the heterogeneous distribution of immune cells in both the axial and lateral directions (**Figure 1G**). Additionally, immune checkpoint genes such as *BTLA, CTLA4, CD96* and *IDO1* were enriched on the tumor side of the interface (19, 113). Similar aggregation of macrophage subpopulations and T cells was also reported in ICC metastases (113). In GBM, a subset of IL-10-releasing *HMOX1*+ myeloid cells were identified in the direct neighborhood of mesenchymal-like tumor regions, which drove T cell exhaustion and thus facilitated the immunosuppressive TME (97). In TNBC, a CD8+ T cell population was identified at the interface instead of tumor core, but their functions need further elucidation (109).

### Treatment response

It is very important to predict the response of cancer patients for specific cancer therapy clinically. However, it is quite difficult due to complex tumor heterogeneity (2, 123). ScRNA-seq has revealed specific cell components could influence treatment response in diverse cancers (127). ST further revealed the specific spatial aggregation and cell-cell interactions of certain cell subpopulations could induce differential treatment responses. For instance, the TLS, which aggregates immune cells in or near the tumor, provides a niche that promotes *in situ* B cells maturation toward plasma cells in RCC (17). These plasma cells disseminate into the tumor tissue and can produce IgG. The tumors with high percentages of IgG-labeled tumor cells were more infiltrated with CD68+ macrophages, which is one of the main effectors of antibody-dependent cellular cytotoxicity (17). This process may promote immunoreactivity and results in better response to ICI treatment. Additionally, the tumor invasive front was found enriched with specific cell subpopulations, which differ in metabolic states and functions from those elsewhere. SAA+ hepatocyte subpopulations near the invasive front are associated with recruitment of M2-like macrophages in ICC, which may form a niche with impaired immune response and promote further tumor invasion and a worse treatment response (19). Moreover, ST also uncovered the connection between specific cell-cell interaction and treatment response. Most *FAP*+ fibroblasts and *SPP1*+ macrophages were colocalized in CRC (90, 121). These *FAP*+ fibroblasts enhanced the recruitment and the proinflammatory activity of *SPP1*+ macrophages through *WNT5A-FZD2* pairs and the expression of TGF-β superfamily genes (immunosuppressive molecules) etc., respectively. In addition, *FAP*+ fibroblasts and *SPP1*+ macrophages upregulated genes of extracellular matrix (ECM)-related pathways, suggesting their role in facilitating the generation of desmoplastic structures, which further limited the immune cell infiltration and induced diminished ICI treatment response (90, 121). Interestingly, the interaction of macrophage subpopulations also affects the treatment response in colorectal cancer liver metastasis (116).

## Clinical application of ST

### Diagnosis

The IHC technique has long been used by physicians and pathologist to diagnose a tissue as benign or malignant, determine the grade and stage of a tumor, identify the cancer cell types, and find the origin of metastasis (128–130). Compared with IHC- and IF-based methods, ST has comparable resolution and is almost transcriptome wide, indicating an enormous potential in cancer pathology (20, 131). Through spot deconvolution and prior cell marker genes, researchers can estimate the cell composition of spots and further divide the ST sections into several spatially different areas i.e., tumor area, tumor leading-edge area and para-tumor area. Several pilot studies have shown ST-based pathological annotations displayed comparable or even higher accuracy than that from pathologists (38, 82, 110, 112, 123) (**Table 2**). Moreover, ST can distinguish cancer subtypes as well (108). For example, Svedlund et al. developed an ISS-based tool called OncoMaps for identification of BC subtypes and predicting recurrence risk (124). Yoosuf et al. trained a machine learning model based on the expert annotation of hematoxylin and eosin (H&E)-stained images and ST data to classify BC tissues into non-malignant, ductal carcinoma *in situ* (DCIS) and invasive ductal carcinoma (IDC) regions with precision up to 96 – 100%. This classification method may provide clinical support for pathologists in the future (125).

### Prognosis associated factors

Prognostic factors can indicate the clinical outcomes of various kinds of diseases (132) (**Table 2**). Currently, three kinds of prognostic factors identified by ST have been reported in multiple cancer types. The first is gene markers. In melanoma, in the tumor compartment, high expression levels of CD8, CD3, TIM3, IDO1 etc. suggested longer progression free survival (PFS), whereas high levels of B2M and PD-L1 in macrophage compartment were associated with longer overall survival (OS) (18). In ICC, hepatocytes close to invasive fronts with high expression level of SAA1 and SAA2 were correlated with worse prognoses (19). Moreover, many gene markers were identified in PDAC, liver cancer, BC, bladder cancer, SCC and GC (53, 92, 112, 114, 121, 126, 133). Cell subpopulations were also potential markers of prognosis. For example, *CCL2*+ endothelial cells and fibroblasts in the deep invasive layer were associated with poor clinical outcomes (91). Similarly, in bladder cancer, CDH12+ epithelial cell was associated with poor prognosis (57). Additionally, *FAP*+ and *INHBA*+ CAFs, and high expression of HSF1 were reported negatively correlated with survival in GC (58, 126). In glioma, blood-derived TAMs indicated poor prognosis (54). A gene signature usually consists of tens to hundreds of genes, which can serve as a prognostic factor as well. For instance, in PC, Taavitsainen et al. proposed a gene signature called PROSGenesis score and further demonstrated high PROSGenesis score was associated with good prognosis (123). In HCC, Wu et al. proposed high TLS-50 signature score was associated with good prognosis (16). Unsurprisingly, higher CAF signature predicted unfavourable PFS and OS in cervical SCC (93) and GC (126).

## Challenges of ST

ST has displayed its advantages in characterizing tumor heterogeneity in gene expression patterns and cell compositions, and potential in clinical studies (12, 134). Though powerful, the cost will limit its wide application. More importantly, two aspects of technical issues also need to concern.

On the one hand, the performance and applicability of ST need to be further improved. First, the detection efficiency of ST is relatively low compared with scRNA-seq (29), which can hardly capture RNA molecules with low expression levels, leading to missing of potential genes that play a role in tumor progression, metastasis, or relapse. Since for most high-resolution SPBC techniques, the area with barcode to capture molecules is less than 30%, thus more than 70% of the mRNA molecules will be missing. Second, most ST approaches only obtain single-end transcripts instead of the full-length transcripts. Thus, it is hard to investigate immune cell receptor repertoires and alternative splicing events, which are important for cancer research. Furthermore, full-length transcripts will make it possible for variant calling across the transcriptome and differential expression analysis at the isoform level as well (135). Third, ST is not exactly suitable for FFPE samples. FFPE tissue blocks, which are the gold standard method of preservation of human tissue for diagnosis, are usually stored for a long time. Consequently, RNA molecules in these blocks are often degraded seriously, hence it is a great challenge to utilize these samples. NanoString DSP and 10x Genomics Visium have shown their compatibility for FFPE tissue blocks (17, 18, 136). However, the quality of data probably varies with specific samples and is much lower than using the fresh one.

On the other hand, the bioinformatic tools for ST data analysis do not meet current needs. Generally, extracting valuable information from raw ST data requires several steps of data processing, including imaging processing, reads mapping, gene expression mapping, followed by downstream analysis such as spot deconvolution, clustering, detection of spatially variable genes, cellular interaction inference, and trajectory inference et al. (137) (**Supplementary Table 1**). Some of these steps, such as deconvolution and clustering, are also frequently used in bulk RNA-seq and/or scRNA-seq data processing, hence many tools initially designed for RNA-seq or scRNA-seq are compatible with ST data. However, their power will unavoidably decrease due to neglect of spatial location and data structure features (50). Thus, it is essential to take features exclusive to ST data into consideration. First, for ST with multicellular resolution, there are plenty of tools for deconvolution of spots, such as SPOTLight (138), SpatialDWLS (139), CARD (140) and Stdeconvolve (141) etc., yet it is difficult to affirm the precise number of cells within a spot but for H&E-stained images. In addition, for ST with subcellular resolution such as HDST (42) and Stereo-seq (43), few tools could identify the individual cell by merging multiple adjacent spots. To distinguish an individual cell from ST is quite important if we want to make full use of this ultra-high-resolution technology. Second, tools for integrating spatial data from multiple batches, platforms, omics, and species are scanty. In this aspect, Spacemake (142), BASS (143) and MAPLE (144) claimed their capacity of multi-platform data integration of ST, but it is essential to further confirm their effectiveness.

Previous studies have shown the prevalent existence of heterogeneity in genomics, transcriptomics, proteomics, and epigenomics (145). However, single-omics technology can only profile the tumor heterogeneity from a specific angle, which ineluctably loses a large amount of information about the other omics. To handle this problem, scientists have developed a series of multi-omics techniques parallel sequencing of single-cell genomes and transcriptomes (146–152). At present, spatial multi-omics technology such as MOSAICA (153), DbiT-Seq (154), SM-Omics (155), NanoString CosMx™ SMI platform (156) and spatial protein and transcriptome sequencing (SPOTS) (157) etc., can quantify the transcriptome and multiple proteins and retain the spatial coordinates. The multi-modal spatial genomics approach provides a promising platform for studying the intrinsic and extrinsic factors contributing to spatial heterogeneity in gene expression and genomic variants (158). Currently, spatial multi-omics has been used in cancer research (41). Another study tried to integrate protein subcellular localization, affinity proteomics, mass spectrometry (MS) data sets and RNA-seq information in a human lymphoma cell line. Their work helps to deepen the knowledge on the architecture of the cells and the complexity of cancer heterogeneity (159). In sum, multi-omics technology has shown its unprecedented value in cancer biology.

## Prospects of ST in cancer research and clinical application

ST techniques have ultrahigh resolution and retain the spatial information of genes and cells, which make them capable of solving many outstanding biological questions. As we know, most current ST can only display gene expression profile and organization of cells on the plane, which is two-dimensional, and does not truly recapitulate the spatial, cellular, and chemical environment of highly complex tumors and their stroma (160). The real spatial atlas should be three-dimensional (3D), which can restore the most realistic spatial environment of cells in tumors, and further provides a more precise atlas and solid basis for research on the mechanism of tumorigenesis and progression, cancer heterogeneity, and clinical applications. Currently, there are bioinformatic tools for reconstruction of 3D ST atlas using multiple sequential adjacent slices and the 3D architecture of human heart (161), cardiac organoid (162), and mouse brain (163, 164) etc. have been reconstructed (**Supplementary Table 1**). However, few studies characterized the 3D structure of tumor tissues (165). We believe the reconstruction and characterization of the 3D architecture of tumors based on ST will revolutionize our knowledge of cancer. In addition, ST may shed light on the role of organelles in cancer. Compared with normal cells, cancer cells display alterations in energy metabolism, which are closely associated with mitochondrial activities (166). Subcellular ST techniques could help to precisely locate the genes that involve in the abnormal energy metabolism and further reveal the mechanism of how they contribute to cancer growth. Previous research also revealed lysosomal related to the dysregulation of tumorigenesis-associated pathways in cancer (166). Interestingly, the intracellular positioning of lysosomes has close connection with the function of cells. For example, the lysosomal subpopulations at the edges of the cancer cell could regulate cell adhesion, exocytosis, and invasion (167). However, it is not clear how the spatial position affects the functions of lysosomes. In this respect, ST techniques such as APEX-seq (47), show great potential for discovering the genes and pathways involved in the functional transition within the organelle. Moreover, ST also shows great potential in premalignant disease research. Currently, the aggressiveness of a premalignant lesion is primarily evaluated by cell morphology (168). However, cell morphology alone is not always sufficient for predicting the evolutionary trajectory of a premalignant lesion. ST remains spatial information as morphology, and it has higher resolution and could predict the differentiation directions of tumor cells based on gene expression profiles and splicing events (169, 170). The additional information provided by ST allows it to identify the precancerous lesions more accurately.

Tumorigenesis, tumor progression and metastasis usually accompany with genomic, transcriptomic, and metabolic changes, which can serve as a basis for diagnosis and identification of subtypes of cancer (171, 172). Recent studies proved ST could obtain these variations (16, 56, 172–175), suggesting ST has great potential in cancer clinical application, especially for digital pathology (DP). In addition, the combination of ST and other information, i.e. pathological imageology and spatial proteasome, may bring new insights into DP (176).

## Concluding remarks

Currently, there are two major kinds of ST techniques in term of the experimental principle, thereinto ISB-based ST technique is among the most promising one due to high resolution and throughput, as well as the ability of whole transcriptome profiling. ST has shown its power in characterizing spatial heterogeneity and clinical applications in cancer. Researchers have profiled the TME in PDAC, BC, CRC, GC, PC, lung cancer, liver cancer, skin cancer, gliomas etc. based on ST. In addition, an increasing number of studies have shown the strength of ST in pathological diagnosis. Many novel potential prognostic markers were also discovered recently. These results jointly suggest ST is a promising technology for comprehensively elucidating spatial heterogeneity, discovering specific spatial structures in tumor tissues as well as for applications in clinical such as pathological diagnosis and prognostic prediction.

With the development of ST techniques and matched bioinformatic tools, the challenge in cost, sensitivity, and automation will be overcome in a few years. In addition, spatial multi-omics technology further integrates transcriptome with other omics such as proteomics, providing more comprehensive landscape of cancer, which may revolutionize our knowledge on cancer. In conclusion, ST technology is progressing rapidly, and it is promising for cancer research and clinical application.

## Author contributions

QY collected the related papers, drafted, and revised the manuscript. LW designed the review and helped to draft and revise the manuscript. MJ prepared the figure and the tables, and participated in the discussion of revision. All authors contributed to the article and approved the submitted version.

## Funding

This project was supported by grants from Shenzhen Key Laboratory of Single-Cell Omics (NO. ZDSYS20190902093613831), Guangdong-Hong Kong Joint Laboratory on Immunological and Genetic Kidney Diseases (NO. 2019B121205005), and Guangdong Basic and Applied Basic Research Foundation (NO. 2021A1515110832).

## Acknowledgments

We thank Dr. Pengfei Qin for constructive advice on this work.

## Conflict of interest

The authors declare that the research was conducted in the absence of any commercial or financial relationships that could be construed as a potential conflict of interest.

## Publisher’s note

All claims expressed in this article are solely those of the authors and do not necessarily represent those of their affiliated organizations, or those of the publisher, the editors and the reviewers. Any product that may be evaluated in this article, or claim that may be made by its manufacturer, is not guaranteed or endorsed by the publisher.
